# Modeling the effective factors on road traffic deaths in Iran 

**Published:** 2019-08

**Authors:** Alireza Razzaghi, Hamid Soori, Amir Kavousi Dolanghar, Alireza Abadi, Ardeshir Khosravi

**Affiliations:** ^*a*^Shahid Beheshti University of Medical Sciences, Tehran, Iran.; ^*b*^Iranian Ministry of Health and Medical Education, Tehran, Iran.

## Abstract

**Background:**

The highest Road Traffic Deaths (RTDs) are related to Low and Middle-Income Countries (LMIC). The efforts for decreasing the incidence and deaths of RTDs can be successful if there is precise information about its related risk factors. This study is aimed at modeling the effective factors of economic, population, road, and vehicles on road traffic deaths in Iran.

**Methods:**

This is an ecologic study which has been done using the covariates of; the proportion of the population, economic growth, urbanization, distance traveled (km) in 100 thousand people, the length of urban roads, the length of rural roads and the number of vehicles for each province in 2015. The regression model of Negative Binomial (NB) was used to modeling these covariates on the deaths of RTDs. The statistical software of STATA edition 14. Was used.

**Results:**

The average of road traffic deaths was 474 (SD= 70.59) in 2015. The result of the multivariate negative binomial model showed that the covariates of the proportion of the population and Gross Domestic Production (GDP) were statistically significant on the number of RTDs.

**Conclusions:**

The covariates of the proportion of population and GDP were effective on the RTDs with the direct and indirect relationship respectively.

**Keywords:**

Death, Road traffic crash, Modeling, Iran

